# Reactive arthritis in the right hip following COVID-19 infection: a case report

**DOI:** 10.1186/s40794-021-00142-6

**Published:** 2021-06-15

**Authors:** Kamyar Shokraee, Soroush Moradi, Tahereh Eftekhari, Rasoul Shajari, Maryam Masoumi

**Affiliations:** 1 Tehran University of Medical Sciences, Tehran, Iran; 2 Student Research Committee, School of Medicine, Tehran University of Medical Sciences, Tehran, Iran; 3 Clinical Research and Development Center, Shahid Beheshti Hospital, Qom University of Medical Sciences, Beheshti Blvd, Qom, Iran

**Keywords:** COVID-19, Spondyloarthritis, Reactive arthritis

## Abstract

**Background:**

SARS-COV-2 usually presents with respiratory symptoms but can have various other manifestations and sequelae. One of the rare complications of COVID-19 infection is Reactive Arthritis. While this complication is more likely to occur following sexually transmitted or gastrointestinal infections, other infections such as COVID-19 can lead to reactive arthritis as well.

**Case presentation:**

Herein, we report a 58 year old woman hospitalized following COVID-19 infection and was discharged after a week. She consequently presented to the clinic ten days after her discharge, complaining of walking difficulties and radiating pain in her right hip. After ultrasound and MRI, she was diagnosed with reactive arthritis inflammation in the hip’s neck. Other known microorganisms responsible for reactive arthritis were ruled out before attributing it to the earlier COVID-19 infection. Clinical symptoms were resolved after being treated using a combination of indomethacin and depot methyl-prednisolone for 14 days.

**Conclusion:**

Latest evidence shows that COVID-19 can lead to autoimmune reactions, including reactive arthritis. Further attention should be paid to symptoms occurring after an episode of infection with COVID-19 to expand our understanding of the disease and the symptoms with which it can manifest.

## Background

Autoimmune reactions to viral and bacterial infections are a known phenomenon, occurring 2–4 weeks after infection and affecting joints in the lower extremities [1, 2]. SARS-COV-2 infection was previously thought to only display respiratory symptoms as part of the *coronaviridae* family [3, 4]; The rapid spread of this virus, however, revealed various sequelae and symptoms, with the autoimmune system having a prominent role in most of these reactions [5, 6]. Herein we report a case of reactive arthritis (ReA) after COVID-19 infection. This case report was prepared following the CARE Guidelines [7].

## Case presentation

A 58-year-old Iranian woman with a previous history of hypertension, coronary heart disease, and type 2 diabetes was admitted to the emergency room complaining of unproductive cough, shortness of breath, and extreme fatigue. RT-PCR test using nasopharyngeal swab yielded positive results for the SARS-COV-2 virus; additionally, CT images showed ground-glass opacity typical of viral pneumonia. Upon hospitalization with an SPO_2_ of 88%, a complete blood workup was performed. She was started on interferon β1, dexamethasone, ceftriaxone, enoxaparin, and nortriptyline and was discharged after 5 days with oxygen levels of 92%. A summary of her laboratory results and her vital information is available in Table 1.

**Table 1:** Laboratory parameters of the patient during hospitalization due to COVID-19 infection

Laboratory Parameter	Day 0	Day 1	Day 3	Day 5
**Mg (mmol/L)**	2.7			
**K (mmol/L)**	4.4	4.3	3.7	4.2
**Na (mmol/L)**	136	136	134	134
**RBC (10**^**6**^**/μL)**	4.55	4.23	4.24	4.65
**Plt (count/μL)**	173,000	262,000	284,000	355,000
**WBC (count/μL)**	4200	7900	6700	10,300
**Hb (g/dL)**	13.6	12.2	12.2	13
**Hematocrit (%)**	37.6	37	36.6	38.1
**MCV (fL)**	82.6	87.5	86.3	81.9
**MCH (pg)**	29.9	28.8	28.8	28
**MCHC (g/dL)**	36.2	33	33.3	34.1
**INR**	1.08			
**PTT (sec)**	44			
**CRP (μg/mL)**	30	44.2	26.2	4.5
**SGOT (AST) (U/L)**	36			
**SGPT (ALT) (U/L)**	38			
**FBS (mg/dL)**	357		224	
**ESR (mm/h)**	83			
**SCr (mg/dL)**	0.9	0.8	0.8	0.7
**Urea (mg/dL)**	41	44	35	40
**ALP (U’L)**	351			
**LDH (U/L)**	778	778	670	623
**PT (sec)**	13			
**Neutrophil (%)**		80	82	
**Lymphocyte (%)**		17	15	

**Fig. 1:**
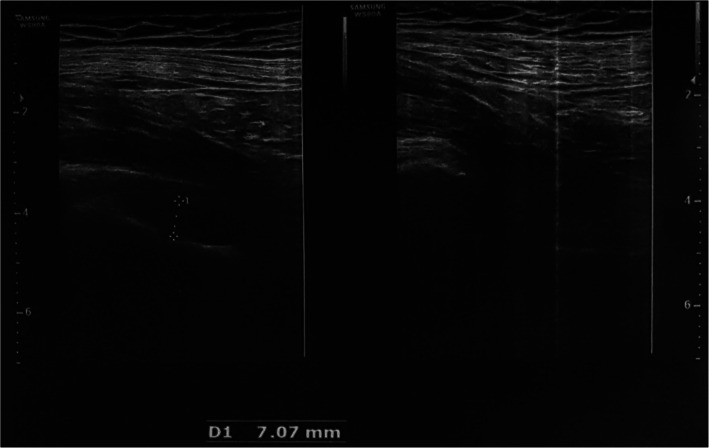
Ultrasonic images of soft tissue around the hip. An increase in thickness is seen in the synovium and also articular effusion in the hip’s neck with a 7 mm diameter

Ten days after her discharge, she represented to the clinic complaining of radiating pain in her right hip, which had caused her walking difficulties. Physical examination revealed a limited range of motion in the right hip due to pain in the right sacroiliac. No redness or warmth was found around the joint, as well as no mouth ulcers, rashes, Raynaud’s, or alopecia. Color doppler sonography of right lower extremities found no signs of either stenosis in the arteries and thrombosis in deep and superficial veins. However, ultrasound images of the soft tissues around the hip revealed an increase in the thickness of synovium and articular effusion in the hip’s neck with a 7 mm diameter (Fig. 1).

**Fig. 2:**
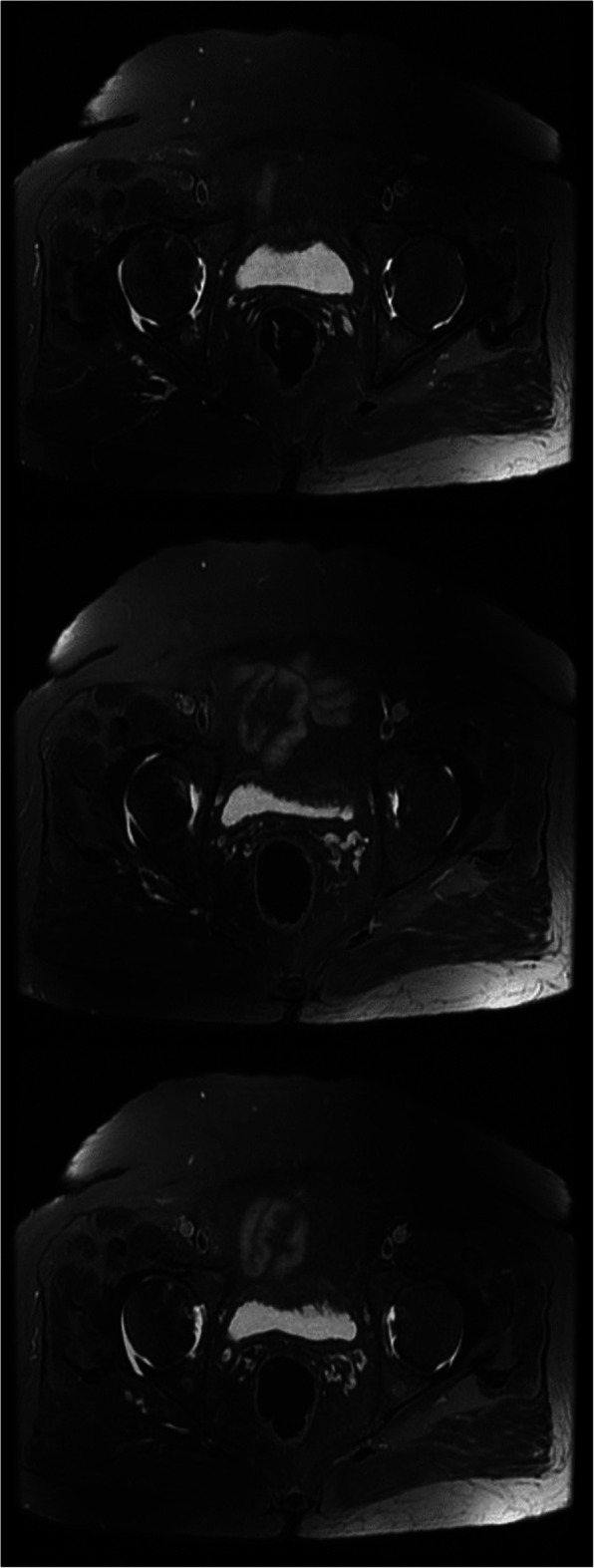
MRI of the pelvis. MRI revealed a fluid rim around the pelvic area, suggesting inflammation in the right hip

Additionally, MRI revealed a fluid rim around the pelvic area, suggesting inflammation in the right hip and sacroiliitis (Fig. 2). When compared to the values recorded at the time of admission for COVID-19 infection, the CRP and ESR of the patient were found to be increased from 5.7 to 6.5 mg/L and from 39 to 45 mmol/h, respectively, which indicated an ongoing autoimmune reaction as a result of the infection. Since reactive arthritis typically happens due to an infection, the patient was tested for Brucellosis (Using Wright, Combs Wright, and 2ME tests) and Tuberculosis (Using PPD skin test) as two of the primary organisms causing ReA. Enteric infections were also symptomatically ruled out. The patient reported no family history of psoriasis, inflammatory bowel disease or autoimmune reactions and had not traveled to any new location in the past 6 months. No genitourinary symptoms which could have suggested a urinary tract infection were reported. IgM and IgG tests of COVID-19 confirmed that the patient had antibodies from her infection with the SARS-COV-2 virus. A diagnosis of possible COVID-19-associated ReA was established based on the lab results and the images.

The patient was started on 100 mg indomethacin twice a day and 80 mg IM depot prednisolone based on this diagnosis. She showed dramatic improvement starting 5 days after her visit and reached remission after 14 days.

## Discussion and conclusions

Reactive arthritis is a form of spondyloarthritis that typically manifests with the involvement of one or a few joints asymmetrically following an infection episode, classically with genitourinary or gastrointestinal microorganisms [8, 9]. These microorganisms, however, are not the only ones to which ReA can be attributed. Previous studies have reported ReA following HIV infection as well as with dengue and chikungunya viruses [10–12]. SARS-COV-2 has been shown to cause multi-organ involvement such as neurologic and gastrointestinal symptoms by triggering autoimmune reactions, leading to reactive arthritis.

Only a few cases of arthritis caused by COVID-19 have been reported until now. Lopez-Gonzales reported 4 cases of acute arthritis during COVID-19 admission, all of which had rheumatologic background diseases [13]. Two other reactive arthritis cases following infection with SARS-COV-2 were reported, one of a 57 years old man in Japan and the other of a 73 years old man in Turkey [14, 15]. In both cases and our case, arthritis symptoms appeared 2–3 weeks after being diagnosed with COVID-19. However, none of these two patients had any symptoms in their hip since their ReA had mainly affected smaller joints, particularly in the hands. To our knowledge, this is the first case of monoarticular ReA in the hip after COVID-19.

Despite our effort to exclude the primary infections responsible for ReA, our ability to perform tests on the patients was heavily limited due to the high amount of resources allocated to COVID-19 patients during the current pandemic. Accordingly, we could not entirely rule out gastrointestinal infections, test the patients for STIs, or obtain a patient’s synovial fluid sample. Further investigation is required to establish the probability of ReA after infection with the SARS-COV-2 and to find the most appropriate treatment for this condition.

## Data Availability

All data of this study are included in this published article.

## Abbreviations

ReA: Reactive Arthritis
RT-PCR: Reverse Transcription Polymerase Chain Reaction
CT: Computed Tomography
MRI: Magnetic Resonance Imaging
PPD skin test: Purified Protein Derivative skin test
HIV: Human Immunodeficiency Virus
2ME test: 2-Mercaptoethanol test
SPO_2_: Peripheral capillary oxygen saturation
RBC: Red blood cell
WBC: White blood cell
Hb: Hemoglobin
MCH: Mean Corpuscular Hemoglobin
MCV: Mean Corpuscular Volume
MCHC: Mean Corpuscular Hemoglobin Concentration
INR: International Normalized Ratio
PTT: Partial Thromboplastin Time
PT: Prothrombin Time
CRP: C-Reactive Protein
ESR: Erythrocyte Sedimentation Rate
SGOT (AST): Serum Glutamic-Oxaloacetic Transaminase (Aspartate aminotransferase)
SGPT (ALT): Serum Glutamic-Pyruvic Transaminase (Alanine transaminase)
ALP: Alkaline Phosphatase
LDH: Lactate Dehydrogenase
FBS: Fasting Blood Sugar
SCr: Serum Creatinine
